# Book Reviews

**Published:** 1892-08

**Authors:** 


### *BOOK REVIEWS.*

THE SCIENCE AND ART OF MIDWIFERY. By William T. Lusk, A. M., M. D. Fourth new and revised edition, 1892. D. Appleton & Co., New York.

The announcement of the publisher of the completion of the fourth edition of Dr. Lusk's book on obstetrics is received with unusual pleasure by the entire medical profession, both in this country and abroad.

Since the publication of the last edition in 1885, such rapid strides have been made in the obstetrical arts and more particularly in the management of the puerperal state as to render the book almost a necessity in the library of every progressive physician. To be well acquainted with the most approved principles of antiseptic midwifery is well worth the price and time occupied in reading this edition. Dr. Lusk may have the proud satisfaction of knowing that he has furnished students and the profession with a work that is a classic among works on the obstetric art.

---

DISEASES OF THE EYE.—A Hand-book of Ophthalmic Practice for Students and Practitioners. By G. E. De Schweinitz, M. D. W. B. Saunders, Philadelphia.

This work of Dr. De Schweinitz has recently made its first appearance before the medical profession. The author, in the opening sentence of his preface, sets forth the intent of the work when he says, "this book has been written in the hope that it may prove of service to students and practitioners who desire to begin the study of ophthalmology." As such, the work, we think, will fully accomplish its object. While the various subjects are in no way treated exhaustively enough to be called a work for reference, yet they are clear and succinct and fully up to all the advancements made in this department of medicine. It is an excellent book for the general practitioner to have in his library and no doubt will soon be adopted as a text-book in many medical schools.

A TEXT-BOOK OF NURSING. By Clara S. Weeks-Shaw. Second edition. D. Appleton & Co., New York.

The literature on the subject of scientific nursing is not very adequate, and hence when a volume like the present one appears, from the pen of one so well qualified to write and teach, its value is readily appreciated. After an experience of several years in the training schools and hospitals of New York Miss Weeks prepared this manual which is certainly now one of the best works of its kind. The contents include directions for the arrangement of the sick room, the observation of symptoms, the preparation of poultices, examination of urine, giving of baths, surgical, medical and obstetrical nursing, control of hemorrhages, care of infants, etc. One of the most valuable chapters is the one on the preparation and administration of foods. Though designed for "training schools and private students" it is a work which will furnish to the active physician a sort of instruction very often needed. It is good reading, and abounds in useful advice and practical suggestions as to the management of the sick, not often found in the books, which is born of patient and intelligent observation at the bedside.

---

THE ELECTRO-THERAPEUTICS OF GYNECOLOGY. By A. H. Gœlet, M. D., Fellow of the New York Academy of Medicine, Vice-President of the American Electro-Therapeutic Association, etc. In two volumes. Geo. S. Davis, Detroit, Michigan.

This work is a description of the author's experience with electricity in gynecology. Dr. Gœlet is one of the most prominent American champions of the methods introduced by Apostoli, though dissenting from the latter on some minor points. The first volume is devoted to a general discussion of electricity and description of apparatus. The second is devoted to therapeutics proper in such conditions as disorders of menstruation, diseases of uterus and appendages, pelvic tumors, ectopic gestation, etc. Dr. Gœlet has brought the electrical treatment in gynecology thoroughly up to date, and those who wish to learn the evidence on this side of an important question will do well to read his book.

A SYSTEM OF GYNECOLOGY. Pozzi. Translated and revised from the French by Curtis M. Beebe, M. D. J. B. Flint & Co., New York, 1892.

This work is a revised translation of the famous French work of Samuel Pozzi, which is the result of his practical experience and hospital services at Louraine. Also based on the most approved and successful methods of the best gynecologists to be found in England, Germany, America and Austria. While the book is condensed, still the translator has not omitted any matter of material importance to the subject under consideration.

The descriptions of pathological conditions and the symptoms necessary for making diagnosis are concise and forcibly put. Also his description of the technique of operations is plain and readily understood by the reader. His methods are those most approved of, and the indications for all operative interference is based on the balance of evidence in favor of a decided benefit to the patient; this is especially noticeable in his treatment of cancers. His views upon the most important of all lesions deserves especial consideration from the profession. I refer to *metritis*. The author considers this as a symptom and thinks it deplorable that some authors confound it with that of a disease. "*Metritis* should remain a clinical term and not an anatomico-pathological one." He also says "from a pathogenitic point of view we may say that all inflammations of the uterus are certainly of infectious origin." The work has justly been placed in the first rank with works on gynecology, and well deserves a place with every man who is interested in the treatment of diseases of women, whether he is a general practitioner or specialist.

W. A. C.

---

THE POCKET PHARMACY, WITH THERAPEUTIC INDEX. By John Aulde, M. D., Member of the American Medical Association, and of the Pennsylvania State Medical Society, etc. New York. D. Appleton & Co.

We must confess that the author's excuse for writing this book, as set forth in the preface, is hardly sufficient. It may supply a "long-felt want," but we fail to see it. It begins with a list of drugs and combinations of drugs suitable to carry in the pocket-case, the *pocket-pharmacy*, and the text of the work is a *résumé* of the clinical application of these remedies in the treatment of emergencies and acute diseases. In this list of twenty-four subjects one may be somewhat surprised at the presence of bryonia, rhus toxicodendron, and trinitrin. The author makes a plea for small doses, and at times approaches uncomfortably near the absurd doctrine of infinitesimals.

